# Associations Between the Density of Oil and Gas Infrastructure and the Incidence, Stage and Outcomes of Solid Tumours: A Population-Based Geographic Analysis

**DOI:** 10.3389/fonc.2021.757875

**Published:** 2021-10-15

**Authors:** Evan Jost, Brittany Dingley, Casey Jost, Winson Y. Cheung, May Lynn Quan, Antoine Bouchard-Fortier, Shiying Kong, Yuan Xu

**Affiliations:** ^1^ Department of Surgery, University of Calgary, Calgary, AB, Canada; ^2^ Departments of Surgery and Oncology, University of Ottawa, Ottawa, ON, Canada; ^3^ University of Calgary, Calgary, AB, Canada; ^4^ Department of Oncology, University of Calgary, Calgary, AB, Canada; ^5^ Department of Community Health Sciences, Cumming School of Medicine, Calgary, AB, Canada

**Keywords:** oil, solid tumour, epidemiology, gas, Geographical Information System (GIS), geography, environment – toxicity, population

## Abstract

**Background:**

We hypothesized that there are geographic areas of increased cancer incidence in Alberta, and that these are associated with high densities of oil and gas(O+G) infrastructure. Our objective was to describe the relationship between O+G infrastructure and incidence of solid tumours on a population level.

**Methods:**

We analyzed all patients >=18 years old with urological, breast, upper GI, colorectal, head and neck, hepatobiliary, lung, melanoma, and prostate cancers identified from the Alberta Cancer Registry from 2004-2016. Locations of active and orphan O+G sites were obtained from the Alberta Energy Regulator and Orphan Well Association. Orphan sites have no entity responsible for their maintenance. ArcGIS (ESRI, Toronto, Ontario) was used to calculate the distribution of O+G sites in each census distribution area (DA). Patient residence at diagnosis was defined by postal code. Incidence of cancer per DA was calculated and standardized. Negative binomial regression was done on O+G site density as a categorical variable with cutoffs of 1 and 30 wells/100km^2^, compared to areas with 0 sites.

**Results:**

125,316 patients were identified in the study timeframe;58,243 (46.5%) were female, mean age 65.6 years. Breast (22%) and prostate (19.8%) cancers were most common. Mortality was 36.5% after a median of 30 months follow up (IQR 8.4 – 68.4). For categorical density of active O+G sites, RR was 1.02 for 1-30 sites/100km^2^ (95% CI=0.95-1.11) and 1.15 for >30 sites/100km2 (p<0.0001, 95%CI=1.11-1.2). For orphan sites, 1-30 sites RR was 1.25 (p<0.0001, 95%CI=1.16-1.36) and 1.01 (p=0.97, 95%CI=0.7-1.45) for >30 sites. For all O+G sites, RR for 1-30 sites was 1.03 (p=0.4328, 95%CI=0.95-1.11) and 1.15 (p<0.0001, 95%CI=1.11-1.2) for >30 sites.

**Conclusion:**

We report a statistically significant correlation between O+G infrastructure density and solid tumour incidence in Alberta. To our knowledge this is the first population-level study to observe that active and orphan O+G sites are associated with increased risk of solid tumours. This finding may inform policy on remediation and cancer prevention.

## Highlights

*Question*: Is there a relationship between oil and gas production facilities and cancer incidence and severity?

*Findings*: Population-level geographic study correlating adjusted incidence of solid tumours with density of active and orphan oil and gas facilities. We found a statistically significant association between cancer incidence and site density for almost all tumour types but did not find any association with mortality or distant metastases.

*Meaning*: This study suggests a correlation between oil and gas production activity and cancer incidence, which provides a basis for further studies on biological and ecological mechanisms for this correlation.

## Introduction

There is increasing recognition of the role of environmental factors in population health. In countries or regions with high oil and gas production such as Canada, this conversation often revolves around petrochemical plants and oil and gas (O+G) infrastructure (1). Oil and gas installations may pose a risk to the health of those who live in close proximity to them (2). However, it is unclear whether living close to these facilities poses a risk for cancer development overall, or whether certain cancer types are more likely to occur.

Several previous studies have noted correlations between residence near or employment at O+G-related sites and increased cancer incidence (3–5). Taken together, these data suggest that there may be a possible link between proximity to petrochemical sites and cancer incidence. However, these studies are limited by analysis of small patient numbers, a single tumour type or a single industrial site. A population-based analysis of multiple tumour types and large numbers of patients in a single region may allow a more robust assessment of these correlations. Moreover, the associations between tumour stage at diagnosis and density of conventional oil and gas facilities are not well studied. Conventional O+G production refers to the drilling, production, and transportation of subsurface oil and gas, as opposed to oil sands or offshore production. Orphan facilities are those for which there is no corporate or individual entity responsible for their operation or remediation. A difference in cancer outcomes (such as cancer-specific survival) among areas with varying densities of O+G infrastructure has not been demonstrated in the literature. Some studies have suggested possible mechanisms for contamination around O+G sites. Possible routes of contamination identified in the existing literature include air contamination or contamination of groundwater (1, 5). We hypothesize that groundwater or air pollution is the most likely mechanism for an association of O+G infrastructure with cancer incidence, although the scope of this study does not encompass identifying this mechanism.

The province of Alberta, Canada, is an excellent study area for such an analysis as both health and O+G related data are available for the same geographic area. We hypothesized that there are areas of Alberta in which a higher incidence of cancer is correlated with increased geographic density of O+G infrastructure. We further hypothesized that facilities that are orphaned or incompletely remediated will have a greater effect on cancer incidence than actively licensed facilities. This study may inform public health efforts and provide information to guide remediation activities in the areas of highest risk.

## Methods

### Study Cohort and Data Sources

This study received ethics approval from the Conjoint Health Research Ethics Board at the University of Calgary. This was a retrospective, population-based geographic analysis incorporating prospectively collected data from the provincial, population-based Alberta Cancer Registry (ACR) and the 2011 Canada census. All adult patients (>= 18 years) who were diagnosed with solid malignant tumours (including breast, colorectal, gastric, lung, pancreatic, head and neck, hepatobiliary, renal, bladder, and prostate cancer) between January 1^st^ 2004 and January 1^st^ 2016 in Alberta were included. Patients who did not have a valid healthcare number were excluded. Patients with multiple cancers were included once based on the first incident cancer. Patient demographics (age, sex, and postal code) and tumour characteristics (such as tumour type and stage) were obtained from Alberta Cancer Registry. Cancer treatment data and patient factors such as comorbidity index are prospectively collected in the ACR. The demographics (age, sex, neighbourhood income level and education levels) of the general population during the same timeframe was retrieved from census data. Patient location was defined using the postal code of residence at the time of diagnosis. Location data for active O+G installations was obtained from publicly available data maintained by the Alberta Energy Regulator (AER), and for orphan oil and gas installations by the Alberta Orphan Well Association (OWA). The OWA data file was accessed on March 3, 2019, and the AER data was accessed May 5, 2019. The OWA is an industry-funded body who takes overall responsibility for orphan installations in Alberta. For the purposes of this study, “sites”, “facilities” and “installations” were all considered synonymous and refer to all O+G infrastructure.

### Statistical Analysis

O+G facility distribution analysis was performed by using the geographic location of each O+G installation provided by the AER and the OWA and plotting these on a base map of Alberta census area polygons obtained from Statistics Canada (Statistics Canada, Ottawa, Ontario). Prior to analysis, the data sources were inspected and non-relevant well and facility types such as water wells were removed. These census areas are known as Distribution Areas (DAs). The DAs have a consistent population contained within them, but different geographic areas. We used ArcGIS Pro to calculate the geographic density of O+G installations in each DA, as the number of installations/100km^2^. Patient locations were separated by postal code, and these postal codes were superimposed on DAs using the Postal Code Conversion File available from Statistics Canada (Statistics Canada, Ottawa, Ontario).

The crude incidence rate of each cancer in each DA was calculated using the number of cancer cases divided by the population at risk. The adjusted incidence rates for each cancer and for all cancers in each DA were calculated using logistic regression (adjusted for age, sex, neighbourhood income level, and education level). The income level is defined as the mean income in a patient’s DA. The cut point used in the regression adjustment is the median income for all cancer patients in the province, which includes our subset. The education level is defined as the proportion of people with high school education or higher in a patient’s DA. The cut point used in regression adjustment is 80%, the median value for all cancer patients in the province. Urban *vs*. rural residence was defined as residence in a municipality with greater than 30,000 population.

Negative binomial regression was performed to determine the association between density of O+G infrastructure and cancer incidence for each DA. The subgroup analysis was conducted for active O+G sites, orphan O+G sites, and total O+G sites, and for each tumour type, respectively. The O+G density (number of O+G facilities/100km^2^) was categorized into three groups: 0, 1-30, and > 30 O+G facilities/100km^2^. 1-30 facilities/100km^2^ was chosen as it encompasses the mean number of facilities per 100km^2^ in areas with O+G infrastructure.

Multivariable logistic regression models were constructed to assess the associations between O+G installation density and patients presenting with stage IV cancer for all cancers and for each tumour type individually. In the multivariable logistic regression model, the co-variates included patient age, sex, rural (*vs*. urban) residence, income, education level, treating institution (academic *vs* non-academic), healthcare zone (Calgary, Edmonton, North, South and Central) and Charlson comorbidity index.

Survival analysis was performed using multivariable Cox regression to investigate the effect of O+G installation density on overall survival (OS) and cancer specific survival (CSS) for all cancer patients. In the Cox regression model, we adjusted patient age, sex, tumour grade, tumor stage, treatment (e.g. surgery, chemotherapy, radiation, and hormone therapy), rural (*vs*. urban) residence, income, education level, treating institution (academic *vs* non-academic), healthcare zone and Charlson comorbidity index.

Maps were produced using ArcGIS Pro 10.6.1 software (ESRI Canada, Toronto, Ontario). All statistical analyses were performed with SAS version 9.4 (SAS Institute, Inc., Cary, NC).

## Results

### Patients and Demographics

Patient demographic data are summarized in **Table 1**. 125,208 cancer patients were included in the study, 46.5% of whom were female. Median age was 66 (IQR=57-75) years. The most common cancers were breast (22%), prostate (19.8%), lung (16.7%), and colorectal (15.7%). Overall, 46.4% of patients died during the follow-up period, of which 36.5% were due to cancer. A total of 27,246 (21.8%) patients were stage IV at diagnosis.

**Table 1:** Patient demographic information.

Variables	Category	Total (N=125208)
Mean age at diagnosis (SD)	Mean (SD)	65.6 (13.2)
Sex (n, %female)	Female	58243 (46.5%)
Adjusted Cancer Incidence (cases/100,000 population) per DA	Mean (SD)	341 (183)
	Median (IQR)	307 (224.8-388.8)
Tumour type	Bladder	3807 (3%)
	Breast	27484 (22%)
	Colorectal	19615 (15.7%)
	Gastric/esophagus	4654 (3.7%)
	Head and neck	4693 (3.7%)
	Liver	3714 (3%)
	Kidney	4729 (3.8%)
	Lung	20868 (16.7%)
	Melanoma	6499 (5.2%)
	Pancreas	4369 (3.5%)
	Prostate	24776 (19.8%)
Stage at Diagnosis	0	2020 (1.6%)
	1	27137 (21.7%)
	2	25586 (20.4%)
	3	10334 (8.3%)
	4	27246 (21.8%)
	Unknown	32705 (2.6%)
Residency area	Rural	31633 (25.3%)
	Urban	93575 (74.7%)
Education (DAs with >=80% high school or greater)	>= 80%	71908 (57.4%)
Income (DAs with median income >= 46,000 CAD/y))	>= 46,000 CAD/y	56374 (45%)
Mortality (n, %)		58111 (46.4%)
Cancer-specific mortality, n (%)		45743 (36.5%)

### Geographic Distribution of Oil and Gas Installations

**Figure 1:**
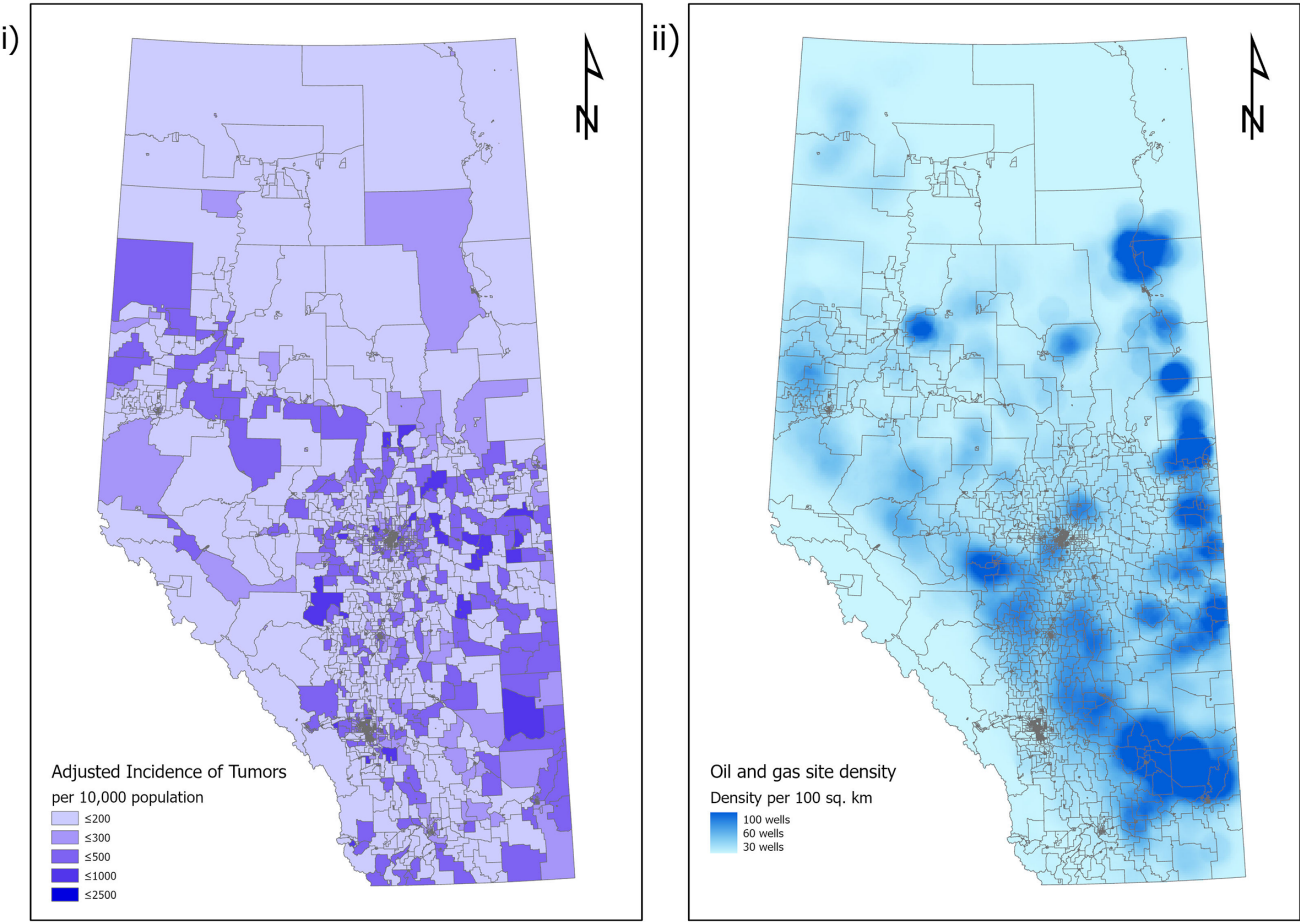
Relationship of cancer incidence in Alberta, Canada to location of oil and gas infrastructure. i) Adjusted incidence of cancer per census distribution area in Alberta. ii) Distribution of oil and gas infrastructure in Alberta.

There were 4,827 DAs and 487,413 O+G facilities in Alberta at the time of data access, with 5,592 (1.1%) orphan sites and 481,821 (98.9%) active sites. The mean number of O+G facilities/100km^2^ in Alberta was 40 (Range 0-231, SD=28), with the median being 0 (IQR=0-0). Most of the DAs with the highest density of installations were in the eastern parts of the province (**Figure 1**). 3921 (81%) of DAs had no O+G infrastructure within them (**Table 2**), the majority of which were in urban areas.

**Table 2:** Patient demographics by facility type and density.

	Total (4827 DAs)	Active sites				Orphan sites				Total sites			
		No well (3292 DAs)	1 - 30/100km^2^ (164 DAs)	>30/100km^2^ (741 DAs)	P	No well (4654 DAs)	1- 30/100km^2^(N166 DAs)	>30/100km^2^ (7 DAs)	P	No well (3921 DAs)	1 - 30/100km^2^ (163 DAs)	>30/100km^2^ (743 DAs)	P
Mean Age (SD)	45.9 (5.2)	45.7 (5.0)	46.2 (6.9)	47.1 (6.1)	<.0001	45.8 (5.2)	48.8 (6.9)	45.8 (2.2)	<.0001	45.7 (5.0)	46.3 (6.9)	47.1 (6.1)	<.0001
Sex (% Male)	50	50	52	51	<.0001	50	52	48	<.0001	50	53	51	<.0001
Education level (DAs with ≥80% of residents with minimum high school education)	3006 (62%)	2554 (65%)	95 (58%)	357 (48%)		2949 (63%)	54 (32%)	3 (43%)		2554 (65%)	95 (58%)	357 (48%)	
Income level (DAs with median income ≥46,000 CAD/yr)	2327 (48.21%)	1883 (48.01%)	95 (57.93%)	349 (47.1%)		2276 (48.9%)	49 (29.52%)	2 (28.57%)		1883 (48.02%)	94 (57.67%)	350 (47.11%)	

### Distribution of Cancer Incidence

Mean adjusted incidence rate of total cancers per DA was 341/100,000 population (range 0-2458, SD=183). Median adjusted incidence rate per DA was 307/100,000 population (IQR=224.8-388.8) (**Table 1**). There was variation in incidence rate among the DAs, with the highest incidence rates in the eastern parts of the province as displayed in **Figure 1**.

### Association of Cancer Incidence With Oil and Gas Facility Density

Results for the association between O+G facility density and cancer incidence can be found in **Table 3**. For all O+G installations when density is treated as a categorical value with zero density being the reference, the Incidence Rate Ratio (IRR) was 1.03 (p=0.43, 95% CI=0.95-1.11) and 1.15 (p<0.0001, 95%CI=1.11-1.2) for a DA with 0-30 facilities/100km^2^ and a DA with >30 facilities/100km^2^, respectively.

**Table 3:** Relationship of O+G site density to cancer incidence, survival, and distant metastases.

	Orphan sites (95% CI, p)	Active Sites (p)	Total sites (p)
Incidence Rate Ratio: continuous	1.2 (0.68-2.11, p=0.52)	1 (1-1.01, p=0.57)	1 (1-1.01, p=0.57)
Incidence Rate Ratio: 1-30 facilities/100km^2^	1.25 (1.16-1.36, p<0.0001)	1.02 (0.95-1.11, p=0.53)	1.03 (0.95-1.11, p=0.43)
Incidence Rate Ratio: >30 facilities/100km^2^	1.01 (0.70-1.45, p=0.9719)	1.15 (1.11-1.2, p<0.0001)	1.15 (1.11-1.2, p<0.0001)
Stage IV at presentation OR: >30 facilities/100km^2^	0.96 (0.69-1.33, p=0.79)	0.94 (0.9-0.99, p=0.0206)	0.94 (0.9-0.99, p=0.0215)
Overall Survival HR: >30 facilities/100km^2^	1.01 (0.87-1.17, p=0.91)	1 (0.98-1.02, p=0.89)	1 (0.98-1.02, p=0.88)
Cancer-specific survival HR: >30 facilities/100km^2^	0.99 (0.83-1.17, p=0.90)	1 (0.97-1.02, p=0.93)	1 (0.97-1.02, p=0.92)

For active sites, habitation in a DA with 0-30 facilities/100km^2^ has an IRR of 1.02 (p=0.53, 95%CI=0.95-1.11) and for >30 facilities/100km^2^ the IRR was 1.15 (p<0.0001, 95% CI=1.11-1.2).

For orphan sites, habitation in a DA with 0-30 facilities/100km^2^ has an IRR of 1.25 (p<0.0001, 95% CI=1.16-1.36) and for >30 facilities/100km^2^ the IRR was 1.01 (p=0.97, 95%CI=0.7-1.45).

The result of subgroup analysis by tumour types showed that increased cancer incidence was associated with higher O+G density (>30 total facilities/100km^2^). These tumours included breast (IRR 1.11, p=0.0058), prostate (IRR 1.11, p=0.021), lung (IRR 1.24, p=0.0001), colorectal (IRR 1.5, p<0.0001), melanoma (IRR 2.12, p<0.0001), renal (IRR 2.39, p<0.001), head and neck (IRR 2.70, p<0.0001), gastric (IRR 1.44, p=0.0022), and hepatobiliary (IRR 1.49, p=0.0077) malignancies (**Table 4**).

**Table 4:** effect of O+G facility density on incidence, metastasis, and survival for individual tumour groups.

	Incidence rate ratio:Continuous (95%CI, p)	Incidence rate ratio: >30 facilities/100km^2^ (95%CI, p)	Stage IV at presentation OR continuous (95%CI, p)	Overall Survival: HR >30 facilities/100km^2^(95%CI, p)	Cancer Specific Survival: HR >30 facilities/100km^2^(95%CI, p)
Breast	1.01 (1-1.02, p=0.27)	**1.11 (1.03-1.19, p=0.006)**	0.98 (0.93-1.04, p=0.51)	1.04 (0.97-1.11, p=0.31)	1.04 (0.96-1.14, p=0.34)
Prostate	1 (0.99-1.01, p=0.74)	**1.11 (1.02-1.21, p=0.021)**	0.99 (0.96-1.02, p=0.43)	0.99 (0.93-1.06, p=0.25)	0.97 (0.89-1.06, p=0.55)
Lung	1 (0.99-1.02, p=0.80)	**1.24 (1.11-1.38, p=0.0001)**	1.01 (0.99-1.03, p=0.27)	0.97 (0.93-1.01, p=0.09)	0.97 (0.93-1, p=0.08)
Colorectal	1.01 (0.99-1.03, p=0.47)	**1.5 (1.34-1.67, p<0.001)**	1 (0.98-1.02, p=0.95)	1.02 (0.97-1.07, p=0.53)	1 (0.94-1.06, p=0.89)
Melanoma	1.03 (0.97-1.1, p=0.31)	**2.12 (1.71-2.64, p<0.0001)**	1.01 (0.98-1.04, p=0.42)	1.03 (0.9-1.18, p=0.64)	1.07 (0.91-1.26, p=0.38)
Renal	1.01 (0.95-1.08, p=0.76)	**2.39 (1.87-3.07, p<0.0001)**	0.98 (0.93-1.03, p=0.40)	0.91 (0.81-1.02, p=0.11)	0.97 (0.85-1.11, p=0.66)
Head and neck	1 (0.98-1.03, p=0.94)	**2.7 (2.11-3.45, p<0.0001)**	1.05 (0.99-1.1, p=0.11)	0.95 (0.85-1.05, p=0.31)	0.99 (0.87-1.12, p=0.87)
Gastric	0.99 (0.93-1.05, p=0.74)	**1.44 (1.14-1.82, p=0.002)**	**1.08 (1.01-1.15, p=0.03)**	1.03 (0.95-1.11, p=0.50)	1.05 (0.96-1.14, p=0.26)
Hepatobiliary	0.96 (0.91-1, p=0.07)	**1.49 (1.11-1.99, p=0.008)**	0.99 (0.92-1.07, p=0.88)	1.05 (0.96-1.15, p=0.28)	1.04 (0.94-1.15, p=0.43)
Pancreatic	1.01 (0.96-1.07, p=0.65)	1.28 (0.98-1.66, p=0.069)	1.03 (0.97-1.11, p=0.31)	0.97 (0.98-1.02, p=0.39)	0.97 (0.89-1.05, p=0.47)
Bladder	0.99 (0.94-1.05, p=0.72)	1.02 (0.77-1.34, p=0.90)	1 (0.83-1.21, p=0.98)	1 (0.9-1.11, p=0.97)	1 (0.88-1.13, p=0.99)

Bolded values are statistically significant.

### Association of Metastasis at Presentation With Oil and Gas Facility Density

For total, orphan, and active facilities, there were no statistically significant correlations between O+G facility density and metastasis at presentation. (**Table 3**).

### Association of Survival With Oil and Gas Facility Density

Survival analysis revealed no negative effect of location near >30 O+G facilities/100km^2^ on overall or cancer specific survival. For active sites, Hazard Ratio (HR) for overall survival (OS) was 1.0 (p=0.89, 95% CI=0.98-1.02) and for Cancer Specific Survival (CSS) was 1.0 (p=0.93, 95% CI=0.97-1.02). For orphan sites, HR for OS was 1.01 (p=0.91, 95% CI=0.87-1.17) and for CSS was 0.99 (p=0.90, 95% CI=0.83-1.17). For total sites, HR for OS was 1.0 (p=0.88, 95% CI=0.98-1.02) and for CSS was 1.0 (p=0.92, 95% CI=0.97-1.02) (**Table 3**). There was no association between O+G facility density and OS or CSS for individual tumour types (**Table 4**).

## Discussion

To date, several studies have reported on cancer incidence in populations residing near industrial sites in various locations. Ghazawi et al. mapped postal code data of over 18,000 Canadian patients and identified a rate of acute myeloid leukaemia greater than three times that of the national average in Sarnia, Ontario, a city known for its numerous chemical plants and oil refineries (3). In a meta-analysis, Wong et al. reported increased incidence of skin cancer in some groups of refinery workers in the UK and upstream oil workers in Canada, although no mechanism for this finding was identified by the authors (4). A systematic review published in 2019 identified three studies which showed excess cancer mortality in oil-extracting regions of Ecuador. They further reported a study performed in Colorado which showed that children with acute lymphocytic leukaemia were 4.3 times as likely as controls to reside near active oil and gas wells (2). A study conducted in Alberta, Canada recognized increased levels of 43 Volatile Organic Compounds (VOCs) in the area downwind of a large petrochemical complex, 10 of which are known, probable, or possible carcinogens. They found increased levels of male hematopoietic malignancies in the same geographic area as compared to surrounding municipalities and the entire province (5).

This study investigated the possible correlation of high densities of O+G infrastructure with cancer incidence. To our knowledge, this is the first study that reports this correlation on a population level in the context of various common solid tumours and different types (active and orphaned) of conventional oil and gas production. The main finding was that cancer incidence was associated with increased density of O+G infrastructure. It is possible that the larger number of active O+G facilities within a DA increases the potential exposure to industrial carcinogens, and therefore increases cancer incidence. This is in keeping with the findings of other studies.

Notably, residing in near areas with orphan wells at low densities was associated with an elevated risk of cancer. This may be due to a lack of appropriate remediation or adequate abandonment and not being actively maintained by any proprietor. This may result in an increase of environmental contamination and therefore increased risk for nearby inhabitants. There is little direct evidence for this, but previous studies have found an increased risk for contamination from orphan and abandoned wells. Kang et al. in 2014 reported increased methane emissions from abandoned oil and gas wells in Pennsylvania, with some of the highest emitters releasing 3 orders of magnitude higher flow rate of methane than the median flow rate of methane for wells in that area (6). We suspect that the reason for the diminished risk ratio in areas of higher orphan well concentration (>30 facilities/100km^2^) is due to the low number of areas with these concentrations. The finding that orphan wells have a stronger association than active wells with cancer incidence may point to an effect of increased contamination near orphan sites, although we have not identified a biological mechanism for this association. This difference in incidence rate ratio is particularly pronounced given the much smaller overall numbers of orphan sites in Alberta.

The association between cancer incidence and O+G facility density was robust among most of the solid tumour types captured in our database. The exceptions to these were pancreatic and bladder cancers. This finding is counterintuitive given that these tumours are diverse in terms of their oncogenesis, risk factors, and clinical behaviour. However, there are several studies which have investigated links between exposure to petroleum products and risk of developing solid malignant tumours. These studies have reported increased risk of rectal, skin, renal, gastric, lung, and prostate cancers in people with long-term occupational or residential exposures to petroleum refineries or products (7–12). These studies, when taken together, suggest a time-dependent risk of oncogenesis in people exposed to hydrocarbons. Studies published by Peters et al. and Kachuri et al. respectively suggested that exposure to diesel and gasoline emissions for periods of greater than ten years would be necessary for increased cancer risk (8, 9).

We did not find an association between metastasis at presentation or cancer specific survival and density of O+G infrastructure. This suggests that even though there is an association with the development of cancer, this is not associated with more advanced disease at presentation or worse survival. We assessed stage IV patients separately because we hypothesized that living near O+G infrastructure might be associated with the development of more aggressive cancer phenotypes which might present at more advanced stages. An Australian industry-wide study of more than 18,000 petrochemical workers found an increased incidence of melanoma, mesothelioma, prostate cancers, renal cancers, and leukaemia, but no excess mortality compared to the wider population (13). This lack of association may reflect the impact of O+G exposures on developing cancers but not on the biology or behaviour of the malignancies once established. Assuming standard treatment according to cancer, stage, and individual patient characteristics, it would be expected that outcomes would be similar to unexposed individuals. While it is possible that these patients had ongoing exposures to environmental contaminants during treatment and recovery, these exposures did not hamper the success of their treatments.

The study has limitations. The common challenge for studies using population-based data is identifying or quantifying individual exposures. We could not assess exposure time for individual patients to determine if their duration of habitation in these areas explained or contributed to the differences in cancer incidence. Another limitation is that we are unable to identify the actual contaminants, if any, to which individual patients are exposed. There are multiple possible environmental contaminants to which patients are exposed and we do not have data to identify which of these contaminants, if any, are enriched in these areas. Some of these contaminants are not related to O+G industry activity, such as radon or vehicle exhaust pollution. This also limits our ability to comment on a biological mechanism for the increase in cancer incidence, although previous studies have identified increased air, water, and soil contamination in proximity to O+G extraction sites (2). We are also unable to control for common carcinogenic exposures and control for the possibility that people work in areas other than their primary residence, which would similarly alter their exposures. We feel that these possibilities are somewhat mitigated by our large number of patients and that the correlation was noted among multiple different tumour types. This is particularly true of the association of breast cancer incidence with O+G site density. Only 15% of O+G field workers are women, and therefore if occupational exposures were the main contributor to increased cancer incidence we would not expect breast cancer to be among the affected tumour types (14). Finally, the use of postal code to geolocate patients is imprecise. Some DAs are geographically large and O+G facilities are not uniformly distributed. Therefore, not all people residing in a DA will have the same risk of exposure to O+G infrastructure.

Despite the limitations, this study is one of the first to identify a significant correlation between residence near O+G infrastructure and cancer incidence. A unique feature of this study is that we were able to identify this correlation at a population level capturing all patients diagnosed with the common solid cancers in our province over 12 years. The large number of patients involved also provides strong statistical validity to our observations. Another advantage of this study is that the geographic area covered by the health administration in our province and the energy regulatory authority is identical. This is a situation which is rare if not unique among petroleum-producing areas. This provides the opportunity to use pre-existing high-quality geographic and health data to explore associations between petrochemical extraction activities and human health.

## Conclusion

In conclusion, this population-level geographic analysis identified a correlation between O+G facility density (active or orphaned wells) and solid tumour incidence. There was no association noted with distant metastasis or survival. There are limitations which reduce our ability to identify which contaminants might be responsible or eliminate potential confounders. These findings may inform future studies to identify specific exposure risks from habitation near O+G infrastructure as well as public health efforts aimed at remediation in our and other jurisdictions.

## Data Availability Statement

The datasets presented in this article are not readily available because the data is protected by privacy legislation in the country of origin and thus cannot be provided without a data transfer agreement. Requests to access the datasets should be directed to EJ, ejost1@jh.edu.

## Ethics Statement

The studies involving human participants were reviewed and approved by Health Research Ethics Board of Alberta-Cancer Committee. Written informed consent for participation was not required for this study in accordance with the national legislation and the institutional requirements.

## Author Contributions

EJ was involved in the conception, design, data analysis, writing, and editing. BD was involved in the conception, design, writing, and editing. CJ was involved in the conception, design, and data analysis. WC was involved in the conception, design, data gathering, and editing. MQ was involved in the conception, design, data analysis, writing, and editing. AB-F was involved in the study conception, design, and editing of the manuscript. SK was involved in the study conception, design, and data analysis. YX is the senior author and was involved in the study conception, design, data analysis, writing, and editing. All authors contributed to the article and approved the submitted version.

## Conflict of Interest

The authors declare that the research was conducted in the absence of any commercial or financial relationships that could be construed as a potential conflict of interest.

## Publisher’s Note

All claims expressed in this article are solely those of the authors and do not necessarily represent those of their affiliated organizations, or those of the publisher, the editors and the reviewers. Any product that may be evaluated in this article, or claim that may be made by its manufacturer, is not guaranteed or endorsed by the publisher.
